# Kikuchi-Fujimoto Disease: A Case of SARS-CoV-2 Infection Triggering the Rare Disease

**DOI:** 10.7759/cureus.35858

**Published:** 2023-03-07

**Authors:** Rita Lencastre Monteiro, Sérgio Cabaço, Leonor Soares, Hugo Inácio, Rodrigo Nazário Leão

**Affiliations:** 1 Internal Medicine, Centro Hospitalar Universitário Lisboa Central, Lisboa, PRT; 2 Internal Medicine, NOVA Medical School, Lisboa, PRT

**Keywords:** sars-cov-2, corona virus disease 2019, necrotizing histiocytic lymphadenitis, kikuchi-fujimoto's disease (kfd), cervical lymphadenopathy

## Abstract

Kikuchi-Fujimoto disease (KFD), or histiocytic necrotizing lymphadenitis, is a rare, benign, and self-limited disease caused by subacute necrotizing regional lymphadenopathy. The etiology is unknown, although virus and autoimmune mechanisms have been proposed. Patients develop enlarged lymph nodes, fever, and, less frequently, extranodal signs. No specific laboratory test contributes to the diagnosis, and lymph node biopsy is the gold standard to define the diagnosis. The treatment is generally with supportive therapy and usually is spontaneously resolved within six months.

In this article, the authors present the case of a 41-year-old female with mild SARS-CoV-2 (severe acute respiratory syndrome coronavirus 2) infection 10 weeks before she was admitted to the emergency department (ED) due to cervical lymphadenopathies and fever lasting over three weeks. Physical examination revealed multiple lymphadenopathies on the submandibular and jugular regions, cutaneous rash, and hepatosplenomegaly. Blood tests showed elevated acute phase proteins, thrombocytopenia, and increased transaminases and lactate dehydrogenase (LDH). Computed tomography (CT) of the neck showed multiple adenopathies at levels I, II, III, and IV according to the Classification for Lymph Nodes from the American Head and Neck Society and American Academy of Otolaryngology on the right side. Excision biopsy was performed and histopathological examination confirmed KFD. A comprehensive analysis performed was unrevealing of an infectious or autoimmune cause and was assumed to be most likely triggered by SARS-CoV-2 infection given the timeframe correlation.

KFD diagnosis is challenging and there are few reported cases of association with SARS-CoV-2 in the literature. Although further investigation is still needed to better understand the relation between them, it is important that physicians take SARS-CoV-2 infection and vaccination into consideration in KFD's differential diagnosis.

## Introduction

Kikuchi-Fujimoto disease (KFD), or histiocytic necrotizing lymphadenitis, is a relatively rare, benign, and self-limited disease caused by subacute necrotizing regional lymphadenopathies [1]. It mostly affects women aged 20-40 years of Asian descent, despite the condition being identified worldwide and in a variety of ethnic backgrounds. Although the etiology is unknown, virus and autoimmune mechanisms have been proposed, and it is thought to have three evolving phases: proliferative, necrotizing, and xanthomatous [2]. Clinical presentation is usually within one to six weeks after SARS-CoV-2 (severe acute respiratory syndrome coronavirus 2) infection, with spontaneous resolution in four to six months.

In 79-94% of cases, KFD presents with enlarged painful lymph nodes, frequently in the posterior cervical region, with the involvement of axillary or supraclavicular areas, usually affecting only one side. In 35-67% of the cases, it is associated with fever. An extranodal extension of the disease includes cutaneous rashes (4-33%), nodules, erythematous papules, and maculopapular lesions, mainly in the face and chest, arthralgia (7-34%) and hepatosplenomegaly (3-15%). Less frequent symptoms include weight loss, nausea and vomiting, headache, night sweats, upper respiratory symptoms, and odynophagia [3]. No specific laboratory tests contribute to the diagnosis since they are usually normal, but the most frequent findings are elevated levels of erythrocyte sedimentation rate (ESR) (79%), elevated C-reactive protein (CRP) (38%) and lactate dehydrogenase (LDH) (53-82%). Less frequently documented are anemia, lymphopenia (64%), thrombocytopenia (5-19%), elevated transaminases (3-8%), and atypical lymphocytes in the peripheral blood (2-5%) [4].

Differential diagnoses should include lymphoma, infectious lymphadenitis, and autoimmune lymphadenopathies, of which the most common is systemic lupus erythematosus. Excisional biopsy is the gold standard exam for the diagnosis of KFD. The diagnosis is histological and shows paracortical areas of apoptotic necrosis with abundant karyorrhectic surrounded by numerous CD68+/myeloperoxidase, histiocytes, CD68+/CD123+ plasmacytoid dendritic cells, and a minority of CD8+lymphocytes and immunoblasts in the absence of neutrophils [4]. KFD’s treatment is based on supportive therapy, and patients usually recover within one to four months. Non-steroidal anti-inflammatories (NSAID) are used, and patients may benefit from corticosteroid therapy, hydroxychloroquine, or intravenous immunoglobulin [5,6] in cases of more severe presentation.

The authors present a case of KFD most likely triggered by SARS-CoV-2 infection.

## Case presentation

**Figure 1:**
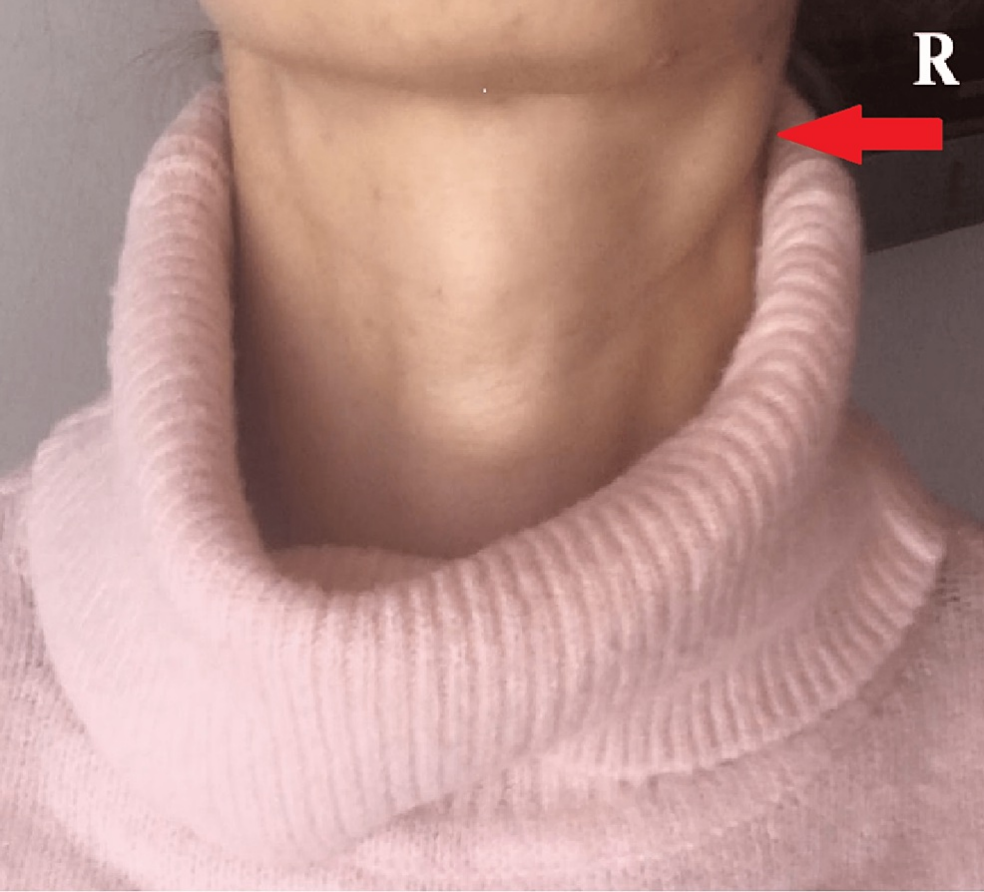
Right cervical submandibular and jugular swelling prominence R – right side. Photograph taken and provided by the patient.

The case presented is a 41-year-old European woman with no personal history of disease, use of regular medication, and no habits of addiction. The patient had been vaccinated against SARS-CoV-2 in the previous year. The patient had a mild form of SARS-CoV-2 infection with myalgias and mild fever for two days. Ten weeks later, she was admitted to the ED due to a three-week painful right cervical swelling. Initially, the pain was referred to only in the submandibular region, but it progressed to the cervical area and worsened by movement. Since the week before, the patient started fever with a maximum axillary temperature of 39ºC. The patient denied night sweats, anorexia, weight loss, adynamia, asthenia, odynophagia, dysphagia, dyspnea, and coughing. She also denied recent personal or family infections and had not traveled recently or had any contact with animals. Physical examination revealed right cervical submandibular and jugular swelling (Figure 1) with multiple lymphadenopathies, enlarged, elastic consistency, non-adherent to deep tissues, and tenderness to touch on the submandibular region and jugular with the largest diameter of 4 cm. There were no other palpable enlarged nodes. The oral cavity showed no alterations. There was a cutaneous rash on the chest and palpable hepatomegaly and splenomegaly in the abdomen.

**Figure 2:**
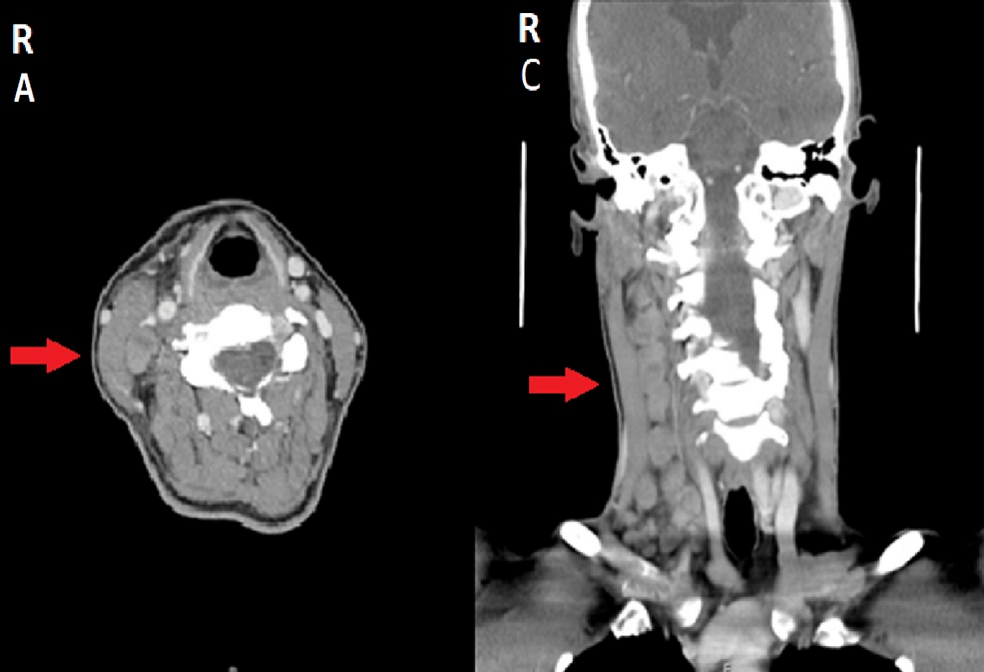
CT of the neck with adenopathies in various ganglionar levels in the right neck R – Right side; A - Axial section CT scan;  C - Coronal section CT scan;  Red arrow points to adenopathy

On admission, she was febrile at 38.6ºC and hemodynamically stable with no other relevant findings. Blood test showed an ESR of 19 mm/h (normal range 0 to 16 mm/h), CRP 22 mg/dL, procalcitonin 0.22 ng/mL, thrombocytopenia 79 × 10^9^/L, increased transaminases (259 U/L alanine transaminase (ALT) and 221 U/L aspartate transaminase (AST)), and LDH 581 U/L, while the remaining parameters were normal. The heterophile antibody test was negative, and the Human Immunodeficiency Virus (HIV), Hepatitis B, and Hepatitis C serologies were negative. The initial autoimmune panel was negative as well as the angiotensin-converting enzyme test. A CT of the neck showed multiple lymphadenopathies at various ganglionar levels in the right neck, including the supraclavicular group, the largest being in the submaxillary topography measuring around 2 x 3 cm, contained in a homolateral submaxillary adenopathic conglomerate with a longitudinal axis of around 6 cm (Figure 2).

**Figure 3:**
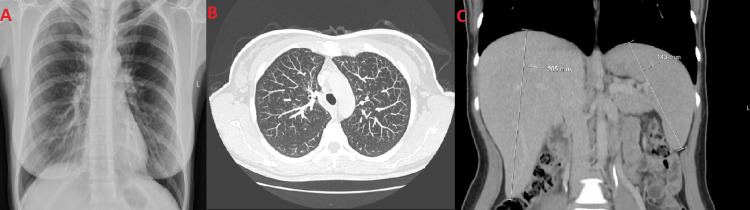
A: Chest x-ray was unaltered; B: Axial CT section of the chest; C: Coronal CT Abdomen showing hepatomegaly of 205 mm and splenomegaly of 143 mm

She was hospitalized and remained febrile (39ºC) within the first few days, which responded to antipyretic medication. Cultural exams did not reveal any isolation. The Interferon-Gamma Release Assay (IGRA) and mycobacterial culture were negative. Cytomegalovirus, Epstein-Barr, *Toxoplasma gondii*, and Parvovirus serologies were negative. Antinuclear antibodies (ANA), anti-double stranded DNA (anti-dsDNA), and anti-Smith antibodies were negative. The chest radiograph was normal as CT-chest did not demonstrate any axillary or mediastinal lymphadenopathy (Figure 3). CT-abdomen revealed hepatomegaly of 205 mm and splenomegaly of 143 mm (Figure 3) and did not present other enlarged lymph nodes.

An excisional biopsy of the right cervical lymphadenopathy was performed throughout the jugular chain and revealed a lymph node with active germinal centers and areas of necrosis, without granuloma formation or neutrophil granulocytes, surrounded by histiocytes (CD68+), T lymphocytes (CD4; CD8) and plasmacytoid dendritic cells (CD123+). No eosinophilic granulocytes, no haematoxylin bodies, and no evidence of lymphoproliferative disease were found. Microorganism investigation by histochemical techniques (Periodic-Acid Schiff (PAS), Grocott and Ziehl-Neelsen) was negative, as well as cytomegalovirus (CMV) investigation by immunocytochemistry. The Acid-Alcohol-Fast Bacilli test, Tuberculosis Nucleic Acid Amplification Tests, and the mycobacteria culture test of lymph node tissue were negative.

The morphological and immunohistochemical alterations favor histiocytic necrotizing lymphadenitis. Considering the course of the disease, laboratory tests, and histopathological examination of the lymph node, KFD was diagnosed, most likely caused by SARS-CoV-2 infection with spontaneous improvement and normalization of laboratory tests. In the follow-up assessment, no relapses were recorded after eight weeks.

## Discussion

KFD is a rare disease and therefore poses diagnostic challenges. The differential diagnosis is based on lymphadenopathy and fever and includes various types of infectious lymphadenitis, lymphoma, and autoimmune lymphadenopathy primarily systemic lupus erythematosus (SLE) [1,7]. Despite many studies, KFD’s etiology has yet to be clearly established. The two main postulated hypotheses are infectious and autoimmune background [7]. The infectious background is the most suggested one, caused by different viral (Epstein-Barr virus, CMV, rhinovirus, rubella virus, and HIV), bacterial, and parasitic pathogens [5,8]. The second hypothesis is an autoimmune background with ANA positivity in a minority of patients. Different connective tissue diseases are associated, mainly SLE (13%) and with some reported cases of polymyositis, rheumatoid arthritis, Still's disease, and Sjogren's syndrome [7-9]. A complete workup, including precise clinical examination with an excisional biopsy, is recommended to rule out other diseases. 

In February 2021, Stimson L. et al [10] published the first case of SARS-CoV-2 infection as the potential etiology of KFD. Some physiopathological mechanisms have been proposed for the development of autoimmunity after COVID-19 (coronavirus disease) as the ability of SARS-CoV-2 to hyper-stimulate the immune system, induction of excessive neutrophil extracellular traps formation with neutrophil-associated cytokine responses, and the molecular resemblance between self-components of the host and the virus [11,12].

The authors reviewed the literature regarding cases of “Kikuchi-Fujimoto disease (KFD)”, “SARS-CoV2” and “COVID-19” by searching medical journal databases written in English in PubMed. Four reported cases of KFD after COVID-19 infection were found [7, 8, 10, 13-15] and another 15 after SARS-CoV-2 vaccination [16-20] were registered until June 2022. Stimson L. et al [10] described the first case of KFD after SARS-CoV-2 infection in February 2021. On the other hand, the first case of KFD after vaccination was reported in March 2022 by Guan et al [16]. Despite KFD’s rare incidence, the growing number of cases reported after SARS-CoV-2 allows us to infer that both entities might be related [20], although further research is needed.

The case described has a typical epidemiological and clinical presentation with fever and cervical lymphadenopathy. It stands out in that cutaneous rash and hepatosplenomegaly are less common. Laboratory results revealed increased inflammatory parameters, thrombocytopenia, increased transaminases, and LDH similar to the cases described in the literature. Spontaneous resolution and no recurrence after three months were observed, with NSAID treatment for a short period of time. At the time of diagnosis, the patient presented no clinical suggestion of infection. Furthermore, the patient did not have a history of autoimmune disease or relevant analytical alterations, so the authors conclude that it is reasonable to assume that a previous COVID-19 infection was the triggering factor of KFD.

## Conclusions

Due to its rarity, clinicians have little contact with KFD. This fact demands high clinical suspicion, and differential diagnosis becomes a real challenge. An incorrect diagnosis can lead to unnecessary procedures and treatments with avoidable complications.

This case strengthens a possible correlation between SARS-CoV-2 and KFD and highlights the importance of taking SARS-CoV-2 infection and vaccination into consideration in the differential diagnosis of KFD, especially in the years after the COVID-19 pandemic.

## References

[1] Perry AM, Choi SM (2018). Kikuchi-Fujimoto disease: a review. Arch Pathol Lab Med.

[2] Hutchinson CB, Wang E (2010). Kikuchi-Fujimoto disease. Arch Pathol Lab Med.

[3] Pepe F, Disma S, Teodoro C, Pepe P, Magro G (2016). Kikuchi-Fujimoto disease: a clinicopathologic update. Pathologica.

[4] Jaseb K, Nameh Goshay Fard N, Rezaei N, Sadeghian S, Sadeghian S (2021). COVID-19 in a case with Kikuchi-Fujimoto disease. Clin Case Rep.

[5] Kucukardali Y, Solmazgul E, Kunter E, Oncul O, Yildirim S, Kaplan M (2007). Kikuchi-Fujimoto disease: analysis of 244 cases. Clin Rheumatol.

[6] Akhila Kavirayani, 25 25 (2017390025). Kikuchi disease with extranodal involvement: response to IVIG and role of inflammatory markers. Rheumatology, Volume 56, Issue suppl_7, December.

[7] Masiak A, Lass A, Kowalski J, Hajduk A, Zdrojewski Z (2022). Self-limiting COVID-19-associated Kikuchi-Fujimoto disease with heart involvement: case-based review. Rheumatol Int.

[8] Al Ghadeer HA, AlKadhem SM, AlMajed MS, AlAmer HM, AlHabeeb JA, Alomran SH, AlMajed AS (2022). Kikuchi-Fujimoto disease following COVID-19. Cureus.

[9] Xu S, Sun W, Liu J (2019). Kikuchi-Fujimoto disease: a case report and the evaluation of diagnostic procedures. BMC Oral Health.

[10] Stimson L, Stitson R, Bahhadi-Hardo M, Renaudon-Smith E (2021). COVID-19 associated Kikuchi-Fujimoto disease. Br J Haematol.

[11] Dotan A, Muller S, Kanduc D, David P, Halpert G, Shoenfeld Y (2021). The SARS-CoV-2 as an instrumental trigger of autoimmunity. Autoimmun Rev.

[12] Cañas CA (2020). The triggering of post-COVID-19 autoimmunity phenomena could be associated with both transient immunosuppression and an inappropriate form of immune reconstitution in susceptible individuals. Med Hypotheses.

[13] Kumar A, Aggarwal V, Sharma S, Singhal A, Jain S, Thakur S (2023). Kikuchi Fujimoto disease and post-SARS-COVID-19 association. Indian J Pediatr.

[14] Racette SD, Alexiev BA, Angarone MP (2021). Kikuchi-Fujimoto disease presenting in a patient with SARS-CoV-2: a case report. BMC Infect Dis.

[15] Iszlai Z, Török L, Tóth E, Karosi T (2022). [Successful management of Kikuchi-Fujimoto disease caused by SARS-CoV-2]. Orv Hetil.

[16] Guan Y, Xia X, Lu H (2022). Kikuchi-Fujimoto disease following vaccination against COVID-19. J Hematop.

[17] Öztürk N, Kılıç İ, Göçün PU, Kaya Z (2022). Kikuchi-Fujimoto disease in a child who had a high suspicion of COVID-19 infection. J Hematop.

[18] Kashiwada T, Saito Y, Terasaki Y (2022). Kikuchi-Fujimoto disease can present as delayed lymphadenopathy after COVID-19 vaccination. Hum Vaccin Immunother.

[19] Tan HM, Hue SS, Wee A, See KC (2021). Kikuchi-Fujimoto disease post COVID-19 vaccination: case report and review of literature. Vaccines (Basel).

[20] Rodríguez-Ferreras A, Maray I, Coya-Fernández C, Octavio-Bocigas MM, Fernández-Del Río MF, Casares-López S, Ruiz-Salazar J (2022). Kikuchi-Fujimoto disease and COVID-19 vaccination: pharmacovigilance approach. Eur Ann Allergy Clin Immunol.

